# Advances in the extraction, purification, structural characterization, and elucidation of the biological functions of polysaccharides from *Hericium erinaceus*

**DOI:** 10.3389/fnut.2025.1634699

**Published:** 2025-08-29

**Authors:** Panling Yu, Jianshuai Ma, Lin Yang, Qin Dong, Changxia Yu, Lei Zha, Baoting Xu, Yan Zhao

**Affiliations:** ^1^Institute of Edible Fungi, Shanghai Academy of Agricultural Sciences, Shanghai, China; ^2^College of Food Science and Technology, Shanghai Ocean University, Shanghai, China

**Keywords:** *Hericium erinaceus* polysaccharides, extraction, purification, structural properties, biological functions

## Abstract

*Hericium erinaceus* has a high food and ornamental value and considerable benefits upon consumption and in research. The high polysaccharide content in *H. erinaceus* gives this fungus unique physiological functions and a smooth, palatable texture; notably, *H. erinaceus* is a superior source of polysaccharides compared with fruits and vegetables. This review focuses on polysaccharides derived from edible mushrooms, an area that has received limited attention in the literature, which has predominantly focused on plant-derived polysaccharides. The relevant literature on the extraction methods, purification processes, structural characterization, and biological functions of *H. erinaceus* polysaccharides (HEPs) has been systematically collated and summarized. *H. erinaceus* polysaccharides have immunomodulatory, lipid-lowering, antioxidant, antitumor, anti-inflammatory, hypoglycemic, and intestinal regulation effects. The aim of this study is to review the research progress pertaining to *H. erinaceus* polysaccharides, providing valuable insights and inspiration for future studies in related fields.

## Graphical Abstract

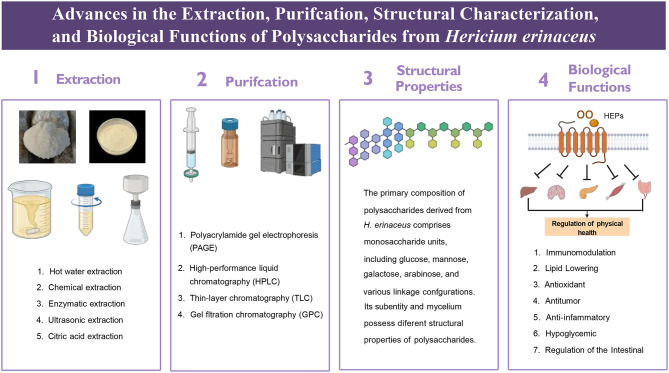

## Highlights

- Methods of extraction and purification of *Hericium erinaceus* polysaccharides (HEPs).
- Different sources of HEPs exhibit distinct structural conformations and characteristics.
- Various mechanisms underlying biological activity and intestinal regulation of HEPs.
- Research challenges and future perspectives of HEPs.

## 1 Introduction

**Figure 1:**
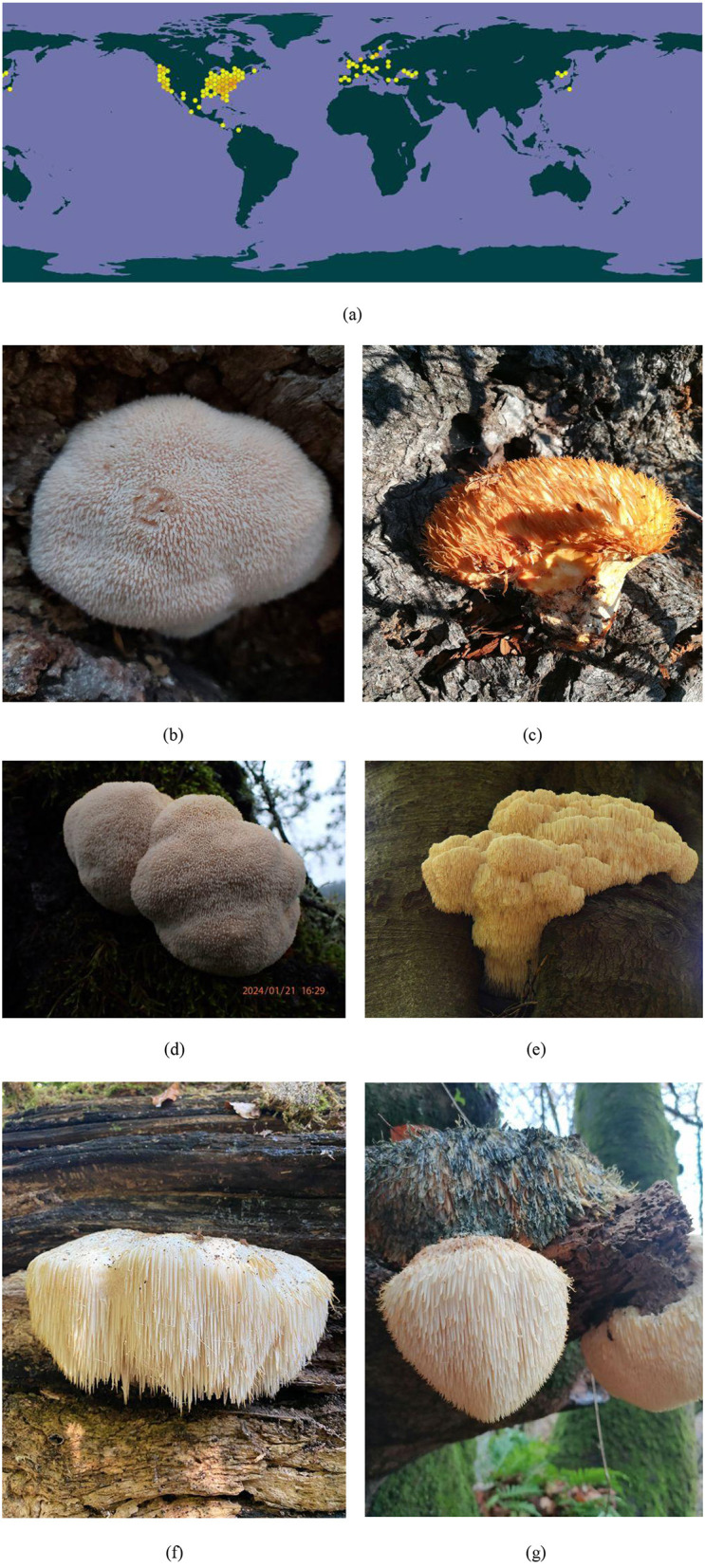
Geographical distribution of *Hericium erinaceus*. **(a)** World map illustrating the distribution of *H*. *erinaceus*. Images from various countries showcasing the different morphological forms of this species: **(b)** China, **(c)** France, **(d)** the United States of America, **(e)** the Netherlands, **(f)** the United Kingdom, and **(g)** Spain (*Source* https://www.gbif.org/species/2537743).

*Hericium erinaceus*, classified within the kingdom Fungi, phylum Ascomycetes, and family Boletaceae, is a notable edible and medicinal mushroom commonly referred to as the monkey head mushroom. Its name is derived from its appearance, which resembles that of a monkey's head. *H. erinaceus* is widely distributed worldwide, with significant prevalence in regions such as China, the United States, and New Zealand. Figure 1 shows the geographical distribution of *H. erinaceus* along with its various morphological forms. The distinct white color and unique shape of *H*. *erinaceus* resemble a cluster of white hairs that gather closely together, further reinforcing its nickname (1). Rich in essential nutrients—including proteins, polysaccharides, fats, vitamins, and minerals—*H*. *erinaceus* is considered a highly nutritious ingredient suitable for culinary applications. In addition to its use as a food ingredient, traditional pharmacology has attributed certain medicinal properties to *H*. *erinaceus*, as it has been utilized to promote human health (2). In recent years, advancements in extraction techniques, combined with an increasing interest in elucidating the mechanisms underlying the pharmacological actions of *H*. *erinaceus* have led to the isolation of various new compounds from both its fruiting bodies and mycelia; consequently, extensive research has been conducted to reveal the pharmacological activities associated with individual constituents. Given that *H*. *erinaceus* has long been employed across Southeast Asia for various purposes—particularly owing to the rising public awareness of health maintenance—*H*. *erinaceus* has attracted high interest worldwide, including in Europe and the United States (3, 4).

*H. erinaceus* polysaccharide is known for its diverse biological activities and nutritional benefits. This complex macromolecule is primarily composed of monosaccharides such as glucose, xylose, mannose, and galactose and features a sophisticated polysaccharide chain structure. Research indicates that *H. erinaceus* polysaccharides exhibit various biological functions, including lipid-lowering, antioxidant, antitumor, immunomodulatory, and anti-inflammatory effects, and these properties contribute positively to human health (5–7). Furthermore, the composition of *H. erinaceus* polysaccharides has been investigated for their influence on the intestinal flora and their potential applications in the regulation of intestinal health. Owing to their bioactivity and nutritional importance, *H. erinaceus* polysaccharides are widely applied in food products, nutraceuticals, and pharmaceuticals. Researchers have comprehensively studied these polysaccharides, including their extraction methods, purification processes, structural characteristics, biological activities, and potential application value. The exploration of *H. erinaceus* polysaccharides not only enhances our understanding of the nutritional profiles associated with this fungus but also provides a theoretical foundation for its applications within the domains of food science and medicine (8, 9). Thus, as crucial bioactive components derived from mushrooms, *H. erinaceus* polysaccharides are abundant in nutrients and exhibit a wide range of biological activities, presenting substantial prospects for utilization in food products, health supplements, and pharmaceuticals. This article discusses recent advancements in *H. erinaceus* polysaccharides worldwide from four perspectives—extraction methods, purification processes, structural characteristics, and biological functions—aiming to provide valuable references for ongoing research into *H. erinaceus* polysaccharides and the development of related products.

## 2 Polysaccharide extraction and purification

### 2.1 Extraction methods

The extraction of polysaccharides from *H. erinaceus* serves as the foundation for investigations into their structure, properties, and pharmacological effects. Currently, various methods, such as hot water extraction, chemical extraction, enzymatic extraction, ultrasonic extraction, and citric acid extraction, have been employed to isolate polysaccharides from *H. erinaceus*, each with distinct characteristics (10). Traditional approaches typically utilize water or organic solvents for extraction; however, the intricate composition of the fungal cell wall complicates the extraction of *H. erinaceus* polysaccharides, potentially altering the biological activity of the extracted polysaccharides. Moreover, conventional techniques are often time-consuming and yield low rates of polysaccharide recovery while frequently necessitating multiple rounds of extraction. The residual liquid produced postextraction can also pose concerns regarding environmental pollution. To address the challenges associated with traditional polysaccharide isolation methods, researchers have turned to advanced techniques, including ultrasonic-assisted extraction, enzyme digestion protocols, and citric acid methods, which markedly increase both the polysaccharide recovery and the extraction yield while minimizing product degradation (11, 12).

The principles and characteristics of various polysaccharide extraction methods for *H. erinaceus* are presented in Table 1. Yang et al. (13) investigated the enzymatic extraction of polysaccharides from *H. erinaceus* substrates via response surface methodology and Box–Behnken design on the basis of one-way and orthogonal experiments to optimize the extraction conditions. The optimal parameters were as follows: pH 5.71, temperature 52.03 °C, and duration 33.79 min. Under these conditions, the polysaccharide yield peaked at 13.46 ± 0.37%, which represents a 67.72% increase compared to the yields obtained after hot water extraction. The extracted polysaccharides were characterized via Fourier transform infrared (FT-IR) spectroscopy, scanning electron microscopy (SEM), circular dichroism (CD), atomic force microscopy (AFM), and gas chromatography (GC) analyses, and the results indicated that the functional groups present in the polysaccharides derived from both the hot water extraction and the enzymatic methods were largely similar; however, notable conformational changes were observed. Furthermore, Wu et al. (14) examined the structural properties and *in vitro* immunological activities of cell wall polysaccharides from *H. erinaceus*, which were extracted continuously using hot water and sodium hydroxide solution. They revealed that the water-soluble cell wall polysaccharides were primarily composed of glucose and galactose, with molar ratios ranging from 3.4:1 to 14:1, but small amounts of glucuronic acid were also detectable within these extracts. The alkali-soluble cell wall polysaccharides consisted predominantly of glucans, which presented lower molecular weights than their water-soluble counterparts but demonstrated a higher macrophage activation activity *in vitro*. Zhang et al. (15) extracted polysaccharides from *H. erinaceus* by several methods and reported that the polysaccharides isolated via ultrasonic extraction exhibited superior performance, demonstrating the highest 1,1-diphenyl-2-dinitrophenylhydrazine DPPH) free radical scavenging activity and providing enhanced protection to L929 cells. Similarly, Yan et al. (16) employed various techniques to extract polysaccharides from *H. erinaceus* and concluded that the citric acid extraction method more effectively preserved the physicochemical properties and biological activities of the polysaccharides. These findings indicate that polysaccharides extracted with citric acid could be developed as bioactive ingredients for both food and medicinal applications.

**Table 1:** Principles and characteristics of different extraction methods.

**Extraction method**	**Principles**	**Characteristics**
Hot water extraction	The elevated temperature of hot water facilitates the breakdown of cell walls, thereby enabling the more efficient release of polysaccharides.	This method is straightforward, cost-effective, and environmentally sustainable. Nonetheless, hot water extraction may result in the degradation of certain heat-sensitive components (8).
Chemical extraction	The polysaccharide is initially dissolved in a chemical solvent, after which the solvent is removed by evaporation or alternative methods to yield the polysaccharide.	Polysaccharides can be extracted efficiently; however, it is essential to select appropriate solvents and operational conditions to prevent damage to the structural integrity of the polysaccharides. Furthermore, the use of solvents may have environmental implications and should therefore be managed with caution (12).
Enzymatic extraction	The enzymatic digestion of polysaccharides from raw materials, facilitated by specific enzymes, enables the release of polysaccharide molecules from their complexes.	Selective extraction of polysaccharides that minimizes structural damage to the raw materials while ensuring relatively gentle treatment (12).
Ultrasonic extraction	The mechanical and thermal effects of ultrasound disrupt the cell wall, thereby enhancing the release of polysaccharides from the raw material.	Ultrasonic extraction has the potential to enhance extraction efficiency, reduce extraction time, and facilitate a straightforward operational process (11).
Citric acid extraction	Complexation: Citric acid, functioning as a complexing agent, can form complexes with metal ions in polysaccharides. This interaction results in a reduction in the bond strength between the polysaccharide and metal ions, which facilitates polysaccharide solubilization and extraction. Acid effect: Citric acid is acidic and can lower the pH of the extraction environment. This decrease in pH is advantageous for disrupting the binding interactions between polysaccharides and other biomolecules, thereby enhancing their dissolution and extractability.	Compared to extraction methods involving strong acids and bases, the use of citric acid for extraction presents notable advantages in terms of mildness. This characteristic facilitates the preservation of both the structural integrity and biological activity of the polysaccharides while simultaneously minimizing their degradation and loss. As a naturally occurring organic acid, citric acid has a less detrimental impact on the environment and aligns with environmental protection standards, thus demonstrating superior ecological friendliness during polysaccharide extraction. Furthermore, citric acid can form complexes with metal ions present in polysaccharides. This interaction aids in eliminating metal ion impurities from the polysaccharides, consequently enhancing their purity. Additionally, as a widely utilized food additive, citric acid is readily accessible and easy to employ, and the citric acid extraction process is relatively straightforward (11).

### 2.2 Purification methods

**Figure 2:**
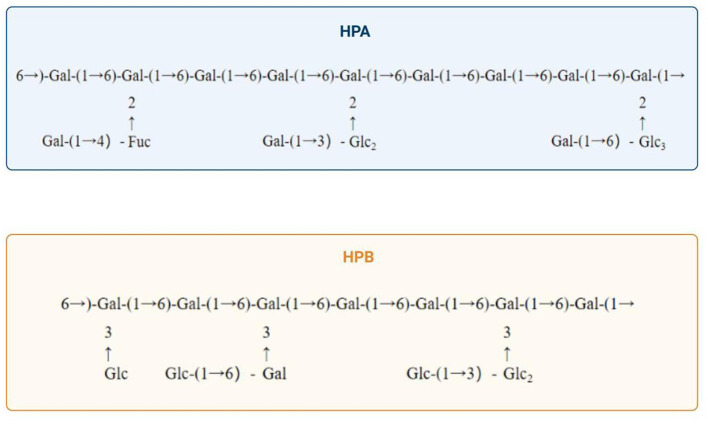
Composition of two *Hericium erinaceus* polysaccharides fractionated and purified via DEAE-Sepharose CL-6B column chromatography (23).

**Figure 3:**
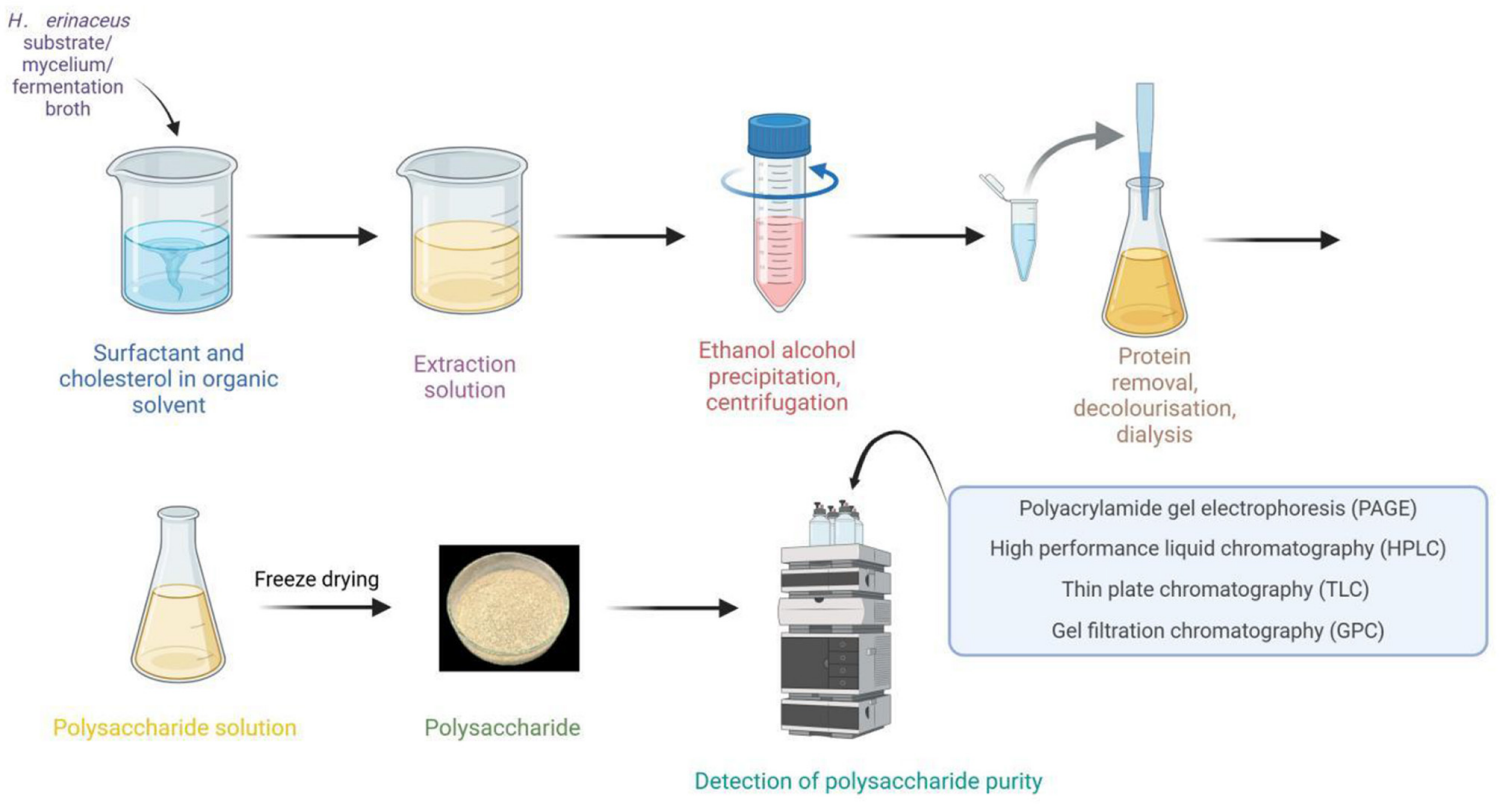
Extraction and purification of polysaccharides from *Hericium erinaceus*.

Purifying *H. erinaceus* polysaccharides is fundamental for investigating their structure and biological activity. The purification process primarily encompasses steps such as deproteinization, decolorization, and graded precipitation. According to the available data, crude polysaccharides exhibit enhanced biological activity following deproteinization and purification (17). Therefore, to increase their activity while also providing purer material for further research, it is essential to deproteinize crude polysaccharides. Moreover, darker colored crude polysaccharides must undergo decolorization; otherwise, measurement errors may increase significantly. Deproteinization can be achieved by several methods, including the Sevag method, the trichloroacetic acid (TCA), and enzymatic methods. Currently, the Sevag method is the most widely employed; however, some studies have demonstrated that the TCA method is more effective, as it results in reduced loss of the polysaccharide content. Enzymatic methods are utilized less frequently because of their high selectivity and the challenges associated with identifying suitable enzymes. Nonetheless, highly efficient deproteinization using compatible enzymes has promising application prospects (18). Decolorization may be achieved using activated carbon, hydrogen peroxide (H_2_O_2_), or diethylaminoethyl (DEAE)-cellulose methods. Oligosaccharides, as small molecules, can be effectively removed by dialysis. Furthermore, polysaccharides can be purified through various methods, including ethanol-grade precipitation, electrophoresis, ultrafiltration, and column chromatography. Commonly employed columns for chromatography include DEAE-cellulose, Sephadex, and Sepharose columns, among others. To assess purity, techniques such as polyacrylamide gel electrophoresis (PAGE), high-performance liquid chromatography (HPLC), thin-layer chromatography (TLC), and gel filtration chromatography (GPC) are predominantly applied (19). Wang et al. (20) utilized hollow fiber ultrafiltration in addition to ion exchange chromatography to purify *H. erinaceus* polysaccharides while eliminating impurities; this approach laid the foundation for subsequent investigations into their polysaccharide activities. Xue et al. (21) extracted a novel polysaccharide from *H. erinaceus* and successfully purified it via DEAE Sepharose CL-6B and Sephadex G-200 column chromatographies for research related to the chelate preparation. Additionally, Qiao et al. (22) isolated an alkali-soluble β-glucan from *H. erinaceus* via hollow fiber ultrafiltration combined with anion-exchange DEAE cellulose column chromatography as well as Sepharose gel column chromatography for purification. Furthermore, Wang et al. (23) fractionated HP—a water-soluble crude polysaccharide derived from *H. erinaceus*—through DEAE-Sepharose CL-6B column chromatography, resulting in two distinct fractions, Hericium polysaccharide A (HPA) and Hericium polysaccharide B (HPB). HPA is composed of glucose, galactose, and fucose at a molar ratio of 1:2.110:0.423, whereas polysaccharide HPB consists solely of the monosaccharides galactose and glucose at a molar ratio of 1:11.529. Upon the application of methylation coupled with gas chromatography–mass spectrometry (GC–MS) analysis and periodate oxidation–Smith degradation and partial acid hydrolysis, the repeat units of HPA and HPB were elucidated, as illustrated in Figure 2; moreover, the HPB extraction and purification protocols are depicted in Figure 3. These methodologies serve as crucial techniques for obtaining high-purity samples of HPB, thereby establishing a solid foundation for subsequent structural characterization and bioactivity assessments.

## 3 Structural properties of polysaccharides

### 3.1 Main structural properties

**Figure 4:**
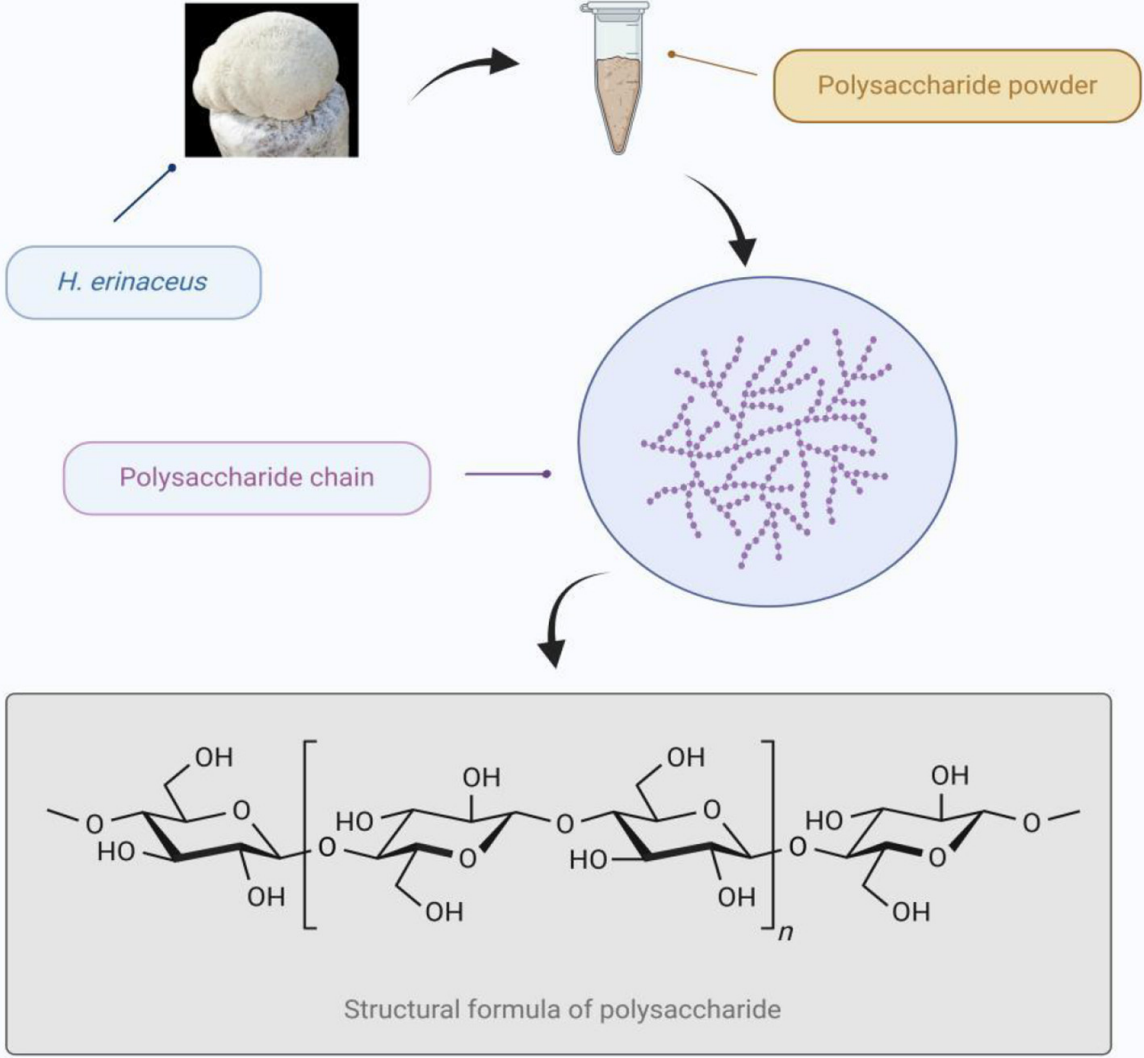
Primary structure of polysaccharides from *Hericium erinaceus*.

Through nuclear magnetic resonance (NMR), infrared (IR) spectroscopy, and mass spectrometry (MS) techniques, researchers have preliminarily analyzed the structural characteristics of polysaccharides derived from *H. erinaceus*. These analyses include the composition of the polysaccharide chains, molecular weights, and molecular structures (24). Investigating these structural properties can provide insights into the mechanisms underlying the biological activities of polysaccharides in *H. erinaceus*. Li et al. (25) predicted the principal structure of these polysaccharides, as illustrated in Figure 4. A variety of polysaccharides with different structures have been isolated and characterized from both artificially cultured and wild *H. erinaceus* substrates, mycelia, and fermentation culture broth. These polysaccharides typically exhibit large molecular weights, ranging from tens to thousands of kilodaltons. Such large molecular weights enhance the stability and biological activity of the polysaccharides *in vivo*; for instance, they play roles in receptor binding and regulating immune function. Additionally, higher molecular weights contribute to the resilience and prolonged effects of polysaccharides within biological systems (26). Polysaccharides derived from *H. erinaceus* are primarily comprised of monosaccharide units, including glucose, mannose, galactose, and arabinose, with various linkages. The specific composition and arrangement of these monosaccharides significantly influence the biological activities and physiological functions of the polysaccharides. For example, combining different monosaccharide units can impact the solubility, stability, and biomolecular interactions of the polysaccharide. Polysaccharides from *H. erinaceus* typically have complex structures consisting of multiple distinct chains that may be linear or branched. The intricacy of this chain architecture plays a crucial role in determining both the biological activity and bioavailability of these polysaccharides. In particular, branching within a structure may influence the solubility and stability, subsequently affecting the rates of degradation and absorption in biological systems. Moreover, the presence of branched architectures can modify how polysaccharides interact with other biomolecules—such as through receptor binding or crosslinking with other polysaccharide molecules (27).

### 3.2 Structural variations in polysaccharides derived from subentities and mycelium

Polysaccharides derived from the subentities and mycelia of *H. erinaceus* exhibit distinct structural characteristics. Polysaccharides isolated from *H. erinaceus* are predominantly heteropolysaccharides, comprising two or more monosaccharides, such as glucose, xylose, rhamnose, mannose, fucose, galactose, and arabinose, with molecular masses ranging from 13 to 1,000 kDa (28, 29). Liu et al. (30) reported the extraction of HEFP-2b, a specific polysaccharide from *H. erinaceus* sourced primarily from its subentity. This polysaccharide is mainly composed of fucose, galactose, glucose, and mannose and has a molecular mass of 32.52 kDa. It features a backbone consisting of ( → 6)-α-D-Glcp-(1 → and → 4)-β-D-Galp-(1 → bonds along with branched units (1 → ) and ( → 6)-β-D-Gap as well as (1 → ) and ( → 4)-α-D-Manp containing glucose and fucose as the residues on the non-reducing end. Moreover, scholars (31) identified a novel polysaccharide from *H. erinaceus* with a molecular mass of 12.714 kDa; this particular substance was predominantly composed of mannose (5.13%), glucose (43.02%), and galactose (51.85%), and its structure consists of various linkages, including (1 → )-Glc, (1 → 4)-Glc, (1 → 6)-Glc, (1 → 6)-Man, (1 → 3/6)-Man, and (1 → 6.-Gal). In contrast to the subentity-derived polysaccharides mentioned earlier, the purified fermented mycelium obtained from *H. erinaceus* yielded mainly heteropolysaccharides along with glycoproteins (32). The monosaccharides that constitute polysaccharides from the mycelia primarily include arabinose, xylose, mannose, galactose, and glucose. Cui et al. (33) isolated an acidic β-glycoprotein with a molecular mass of 14.4 kDa and a protein-to-polysaccharide ratio of 10:1 from *H. erinaceus* mycelium. This compound contains D-glucose, L-rhamnose, D-galactose, and D-mannose, and its main chain is composed of (1 → 4)-linked galactose and glucose residues. Shao et al. (34) identified a novel polysaccharide, EP-1, with a molecular mass of approximately 3.1 kDa that features an α-D-Glc-(1 → 3) backbone structure along with β-D-Glc-(1 → 3) branches at the C-4 position with α-D-Gal-(1 → 3) side chains; the terminal residue is α-D-Man.

## 4 Biological functions of polysaccharides

Studies have demonstrated that polysaccharides derived from *H. erinaceus* exhibit considerable antioxidant properties and can effectively scavenge free radicals. Furthermore, these compounds positively affect human health by reducing blood lipid and glucose levels, demonstrating antitumor effects, modulating the immune system, and regulating the intestinal microbiota (35). These bioactivities provide a crucial scientific foundation for the potential development of *H. erinaceus* polysaccharides as natural pharmaceuticals or health products.

### 4.1 Immunomodulatory effects

Immunity refers to the process by which the body identifies, combats, and eliminates foreign substances (e.g., pathogenic microorganisms, viruses, and bacteria) as well as abnormal cells (e.g., tumor cells and senescent cells) through the immune system. The primary function of immunity is to protect the body against infections, diseases, and foreign agents (36). Polysaccharides derived from *H. erinaceus* play crucial roles in modulating immune system function. These polysaccharides can activate various defense cells—including macrophages, T lymphocytes, B lymphocytes, cytotoxic T lymphocytes, and natural killer (NK) cells—while also facilitating the expression of biochemical compounds such as cytokines and chemokines that exhibit innate antiproliferative properties. Furthermore, these components can induce apoptosis in tumor cells and disturb the differentiation of tumor cells while promoting the secretion of reactive nitrogen species, oxygen intermediates, interleukins, and other bioactive substances. Several studies have demonstrated that *H. erinaceus* polysaccharides can stimulate splenic lymphocytes and macrophages while regulating and enhancing lymphocyte immune functions by modulating signaling pathways such as the nuclear factor kappa B (NF-κB), mitogen-activated protein kinase (MAPK), and phosphoinositide 3-kinase/Ak strain transforming (PI3K/Akt) pathways (35, 37). Immunofluorescence staining demonstrated that the enzymatic products of *H. erinaceus* polysaccharides significantly enhanced the phagocytic activity of nitric oxide (NO), cluster of differentiation 40 (CD40), and CD86 in macrophages, thereby augmenting their immunomodulatory functions in cyclophosphamide-induced immunosuppressed mice (38). Additionally, experiments on the mouse macrophage line RAW264.7 and the human intestinal epithelial cell line cancer coli-2 (Caco-2) revealed that *H. erinaceus* polysaccharides promoted the production of NO, interleukin-6 (IL-6), interleukin-10 (IL-10), and tumor necrosis factor-alpha (TNF-α), leading to increased immune activity (39). Furthermore, applying micro- and nanotechnology to *H. erinaceus* polysaccharides or their modification with specific molecules may enhance their immunomodulatory effects through targeted delivery and improved intestinal permeability. For example, encapsulating *H. erinaceus* polysaccharides in multiwalled carbon nanotubes (MWCNTs) effectively modulated the immune response in mice by significantly increasing immunoglobulin levels and promoting splenic lymphocyte activation (40). Similarly, selenide-encapsulated *H. erinaceus* polysaccharides formulated in poly(lactic acid-hydroxyacetic acid) copolymer nanoparticles not only enhanced the immunoreactivity of these polysaccharides but also significantly increased their phagocytosis by macrophages and upregulated CD40 and CD86 (41).

### 4.2 Lipid-lowering effects

Hyperlipidemia is characterized by abnormal or disturbed lipid metabolism, which increases the risk of cardiovascular diseases, central nervous system disorders, and other health issues. Consequently, the development of safe and effective lipid-lowering agents has a high social and economic implications and has emerged as a focal point of research worldwide. Although contemporary clinical lipid-lowering medications have substantially contributed to human health, they are also associated with adverse effects such as liver dysfunction, pancreatic impairment, and nervous system damage (42). As a result, interest in natural food-derived bioactive compounds for combating metabolic disorders is increasing.

**Figure 5:**
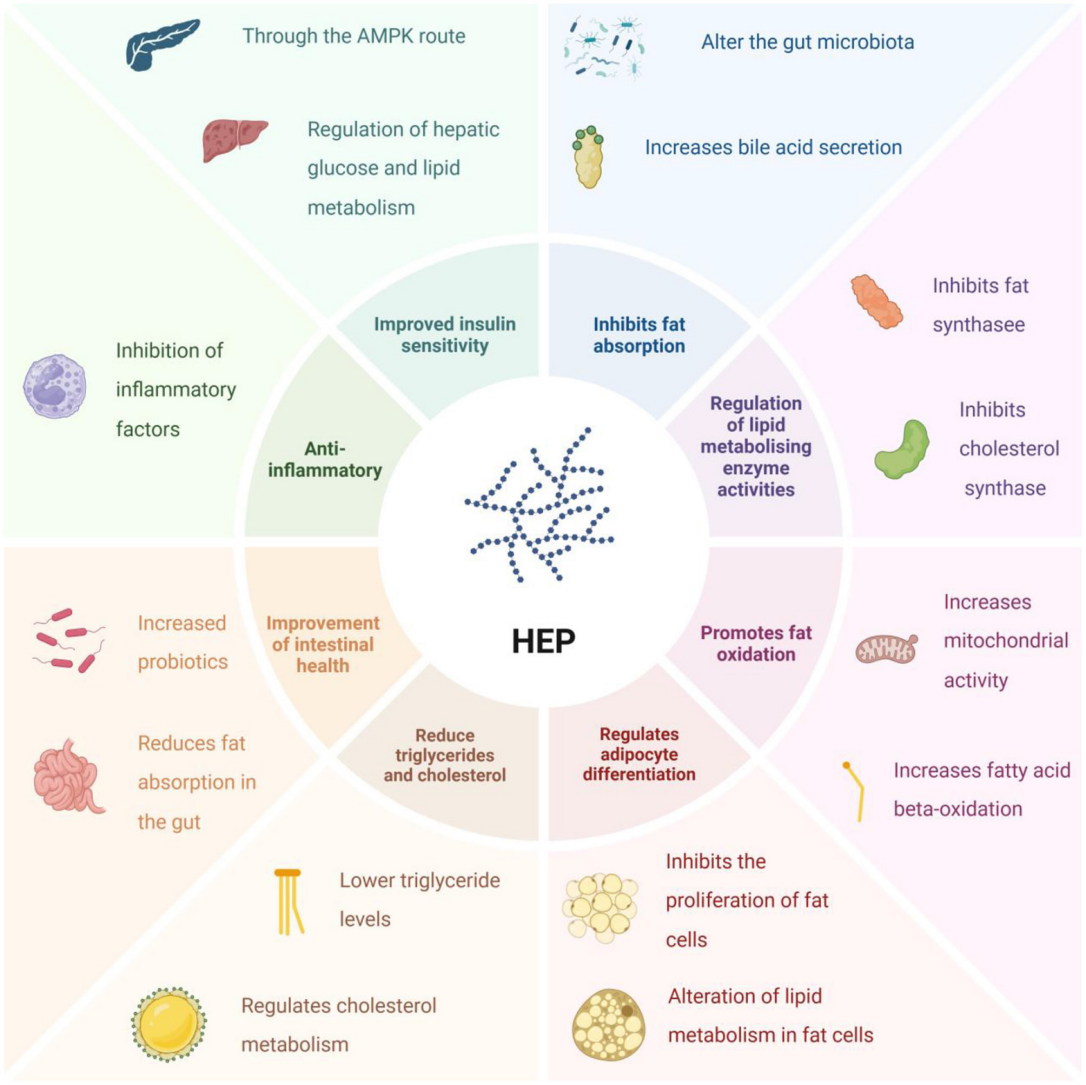
Lipid-lowering effects of polysaccharides from *Hericium erinaceus* (HEP: *H. erinaceus* polysaccharide).

Research has shown that polysaccharides derived from *H. erinaceus* can play a significant role in lowering blood lipids. These polysaccharides influence lipid metabolism through various pathways, as illustrated in Figure 5. This includes the regulation of hepatic cholesterol synthesis and the promotion of cholesterol excretion and metabolism, ultimately leading to reduced blood cholesterol levels. Such regulatory effects are crucial for the prevention and management of hypercholesterolemia. Furthermore, polysaccharides from *H. erinaceus* may offer benefits in terms of preventing conditions such as obesity and fatty liver by inhibiting adipocyte formation and reducing the activity of lipogenic enzymes, thus diminishing the synthesis and storage of fat within the body (42). Additionally, their antioxidant properties enable these polysaccharides to mitigate oxidative stress-induced damage to vascular endothelial cells, thereby helping to prevent the formation of atherosclerosis. Consequently, this contributes significantly to lowering blood lipid levels, particularly low-density lipoprotein cholesterol (LDL-C) levels. Moreover, *H. erinaceus* polysaccharides may enhance blood circulation by regulating vascular tone and promoting vasodilation, thereby assisting in reducing blood lipid levels while simultaneously decreasing the risk of atherosclerosis. Yang et al. (43) successfully isolated an extracellular polysaccharide with notable hypolipidemic effects from liquid cultures of *H. erinaceus*, which presented a protein content of 8.8%, a molecular weight of less than 40 kDa, and a sugar content of 91.2%. Feng et al. (44) investigated the mechanism by which β-glucan inhibits starch digestion in *H. erinaceus*. They analyzed how β-glucans with different molecular weights interact with starch to inhibit its digestion. The rates of starch digestion and glucose release decreased as the molecular weight of the *H. erinaceus* β-glucan increased. Notably, *H. erinaceus* β-glucan attenuated the leaching of straight-chain starch while simultaneously increasing the peak, minimum, and final viscosities. Furthermore, *H. erinaceus* β-glucan was shown to encapsulate starch particles via laser confocal scanning microscopy. *H. erinaceus* polysaccharides (HEPs) can promote bile acid secretion by regulating the intestinal microbiota. Bile acids effectively remove fats and help the liver convert excess cholesterol into bile acids for excretion, thereby reducing bad cholesterol (LDL-C) in the blood. Additionally, the β-glucan in HEP can coat starch particles in food, forming a protective layer around them, thereby slowing down their digestion into glucose and reducing fat synthesis (Figure 5). It both promotes fat excretion and reduces new fat production, and this dual action is key to its lipid-lowering effect. Therefore, it can be inferred that the regulation of glucose release and starch digestion by *H. erinaceus* β-glucans may be attributed to its capacity to increase system viscosity and enhance starch coating, which collectively contributes to a hypolipidemic effect.

### 4.3 Antioxidant effects

Human cells derive energy through biological oxidation reactions to maintain the body's normal physiological functions. Concurrently, the formation of free radicals within cells can lead to structural and functional damage, resulting in various conditions, such as heart disease, cancer, diabetes, and immune system disorders. The excessive generation of reactive oxygen species (ROS) or insufficient antioxidant capacity within an organism triggers a cascade of lipid peroxidation reactions that compromise cell membranes, ultimately leading to cell death and accelerating aging. *H. erinaceus* polysaccharides exhibit considerable antioxidant activity and can scavenge free radicals, thereby reducing oxidative stress-related damage in the body. This contributes to maintaining intracellular homeostasis, mitigating the effects of aging, and providing protection against diseases associated with oxidative stress. At certain concentrations, *H. erinaceus* polysaccharides have various effects on scavenging DPPH free radicals, hydroxyl radicals, and superoxide anion radicals, and their scavenging effects are comparable to those of vitamin C (45, 46). Moreover, Tian et al. (7, 47) reported that *H. erinaceus* polysaccharides effectively increase the total antioxidant capacity (T-AOC) and the glutathione peroxidase (GSH-PX) and total superoxide dismutase (T-SOD) levels while reducing the malondialdehyde (MDA) content, thus increasing the antioxidant capacity in the hepatic tissues of mice. Xu et al. (48) investigated the pharmacological properties of *H. erinaceus* polysaccharides in aged rats and reported that these compounds significantly enhanced skin antioxidant enzyme activities as well as the levels of matrix metalloproteinase-1 (MMP-1), tissue inhibitor of metalloproteinases-1 (TIMP-1), and collagen in a dose-dependent manner. In conclusion, it can be inferred that *H. erinaceus* polysaccharides possess antiskin-aging activity.

### 4.4 Antitumor effects

**Figure 6:**
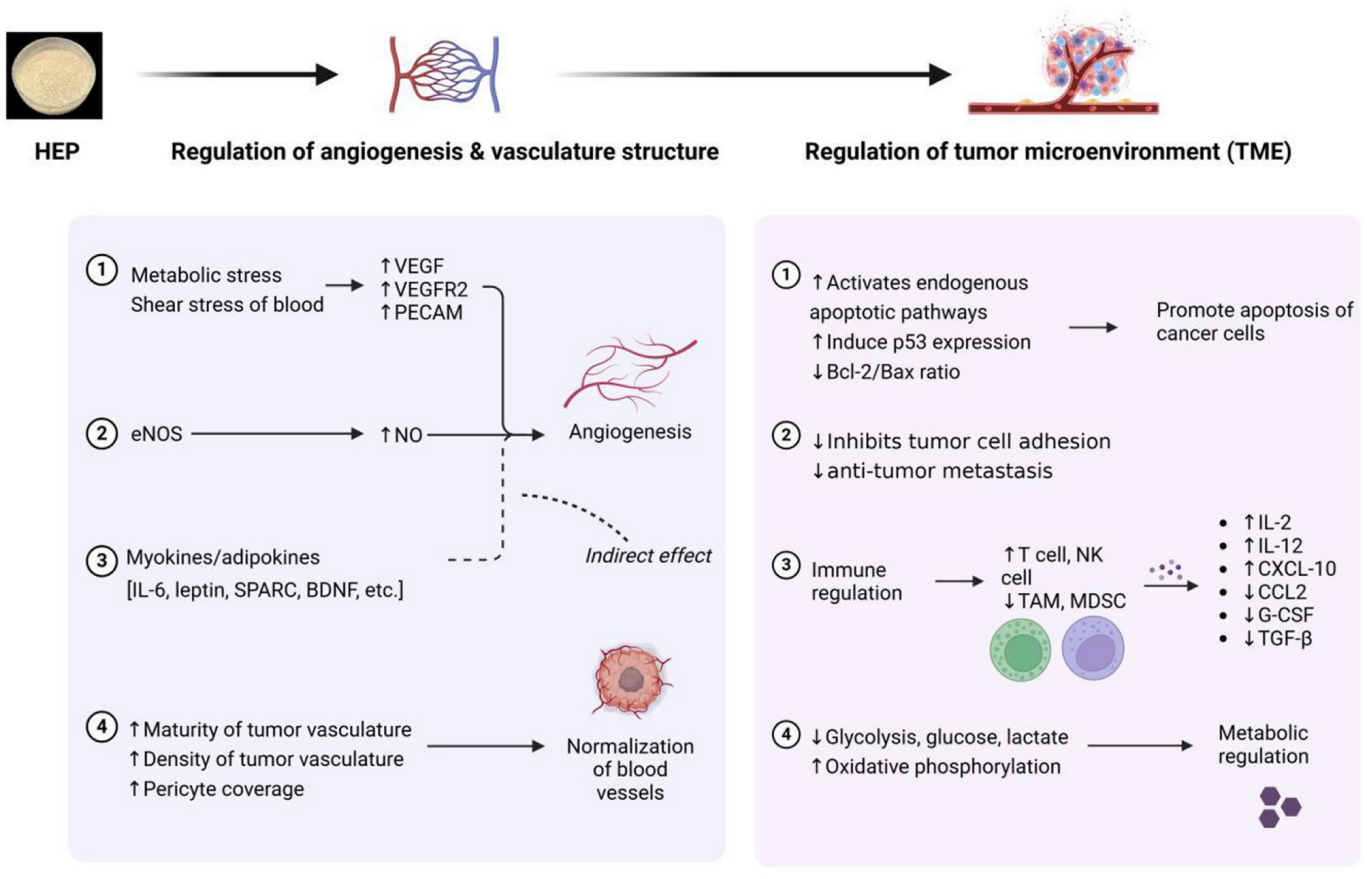
Primary mechanisms of the antitumor activity of polysaccharides from *Hericium erinaceus* (HEP: *H*. *erinaceus* polysaccharide).

The continuous deterioration of the environment and increasing pressures on daily life have made cancer one of the leading causes of mortality worldwide. Tumors arise from the abnormal differentiation and development of cells, which grow uncontrollably within an organism. Traditional surgical interventions and radiotherapy often damage healthy tissues; consequently, natural active antitumor substances are emerging as promising areas for research. Polysaccharides derived from *H. erinaceus* exhibit inhibitory effects on various tumor cell types; they can induce apoptosis, inhibit proliferation and metastasis, and exert regulatory effects on the tumor microenvironment. Thus, these polysaccharides hold high potential for application in both the prevention and treatment of tumors (49). The primary mechanisms underlying the antitumor effects of *H. erinaceus* polysaccharides are illustrated in Figure 6. The antitumor activity of HEPs is primarily achieved through two mechanisms: first, polysaccharides can activate the cell apoptosis program. Polysaccharides bind to the Dectin-1/Toll-like receptor (TLR4) receptors on the surface of tumor cells, initiating signal transduction, increasing the expression of pro-apoptotic proteins Bax/Bad, and simultaneously reducing the expression of antiapoptotic proteins Bcl_2_. This ultimately activates the “scissor protein” cysteine-aspartic acid protease 3 (Caspase-3), leading to the self-destruction of tumor cells. On the other hand, polysaccharides disrupt the tumor's energy supply. By inhibiting the PI3K/Akt pathway (similar to turning off the cell survival switch), polysaccharides block the energy supply required for tumor cell proliferation, trapping them in the S phase and preventing division. Kim et al. (50) demonstrated that *H. erinaceus* polysaccharides interact with the membrane of tumor cells to exert an inhibitory effect while also effectively inhibiting artificially induced lung metastases. Furthermore, Zan et al. (51) reported for the first time that HEG-5, a purified glycoprotein extracted from the fermented mycelium of *H. erinaceus*, significantly inhibits apoptosis and induces S-phase arrest in SGC-7901 cells by promoting their proliferation and colony formation. The results obtained from reverse transcription–polymerase chain reaction (RT–PCR) and protein blotting analyses indicate that glycoproteins significantly reduce the expression levels of Bcl2, PI3K, and AKT1 while concurrently increasing the expression of Caspase-8, Caspase-3, p53, CDK4, Bax, and Bad. These findings suggest that the molecular mechanisms underlying the apoptosis and cell cycle arrest induced by purified glycoproteins derived from fermented *H. erinaceus* mycelia are mediated through Caspase-8/-3-dependent pathways that are independent of p53, mitochondrial involvement, and the PI3K/Akt signaling pathway. Consequently, this study provides compelling evidence supporting the potential utility of purified glycoproteins from fermented *H. erinaceus* mycelia as promising drug candidates for gastric cancer treatment.

### 4.5 Anti-inflammatory effects

**Figure 7:**
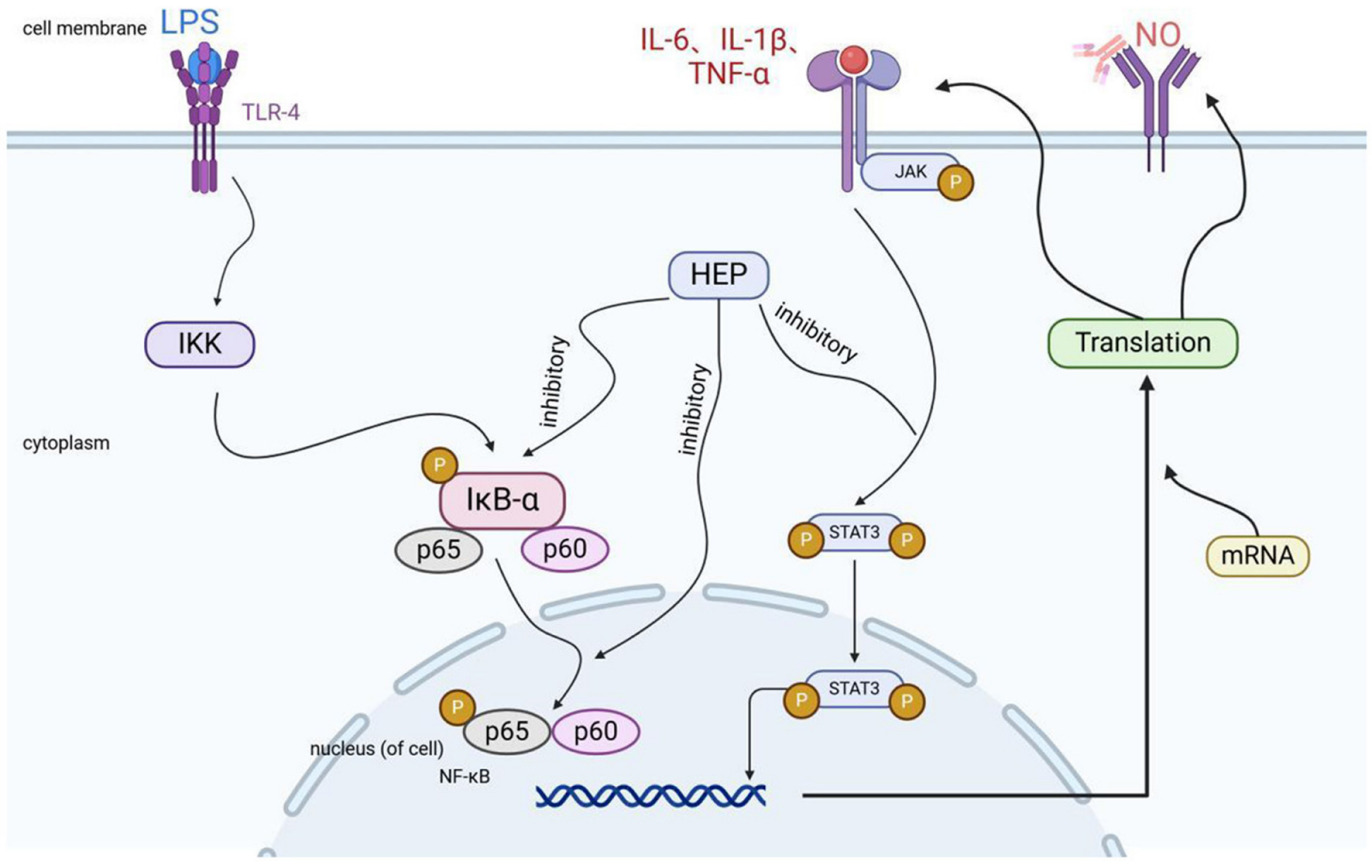
Mechanisms of the anti-inflammatory effects of *Hericium erinaceus polysaccharide* (HEP: *H. erinaceus* polysaccharide).

Inflammation in an organism is characterized by fever, which is a normal physiological response of the immune system to external stimuli such as pathogenic microorganisms. This process is regulated by various inflammatory factors. Mild inflammation plays a crucial role in enhancing the body's immunity and inhibiting the proliferation of bacteria, viruses, and other microorganisms; however, excessive immune stimulation can lead to a significant release of inflammatory factors, resulting in organ inflammation or even organ failure (52). The polysaccharide derived from *H. erinaceus* has been shown to inhibit inflammatory reactions and alleviate symptoms associated with inflammation-related diseases. Its potential value for preventing and treating inflammatory disorders is notable, and its anti-inflammatory mechanisms are outlined in Figure 7 (53). When bacterial toxins (such as LPS) activate inflammatory signals, HEPs can directly block the progression of two pathways, namely, the NF-κB and JAK-STAT pathways. This reduces the production of inflammatory factors (such as TNF-α and IL-6). In neuroinflammatory models, HEPs also significantly reduce the levels of inflammatory factors (NO and ROS) in the brain, thereby protecting neurons from damage. Kushairi et al. (54) explored the anti-inflammatory properties of *H. erinaceus* polysaccharide and demonstrated its neuroprotective effects against hydrogen peroxide-induced neurotoxicity in mouse hippocampal neurons (HT22 cells). Additionally, this polysaccharide significantly reduced NO levels in lipopolysaccharide (LPS)-treated microglia (BV2 cells), suggesting that it has anti-inflammatory effects. Further research conducted by Zhang et al. (55) revealed that sulfated *H. erinaceus* polysaccharides exhibited strong anti-inflammatory effects *in vitro* by regulating the protein levels of NF-κB p65, STAT1, and TLR4 via the NF-κB and Janus kinases—signal transducers and activators of transcription 1 (JAK-STAT1) signaling pathways.

### 4.6 Hypoglycemic effects

**Figure 8:**
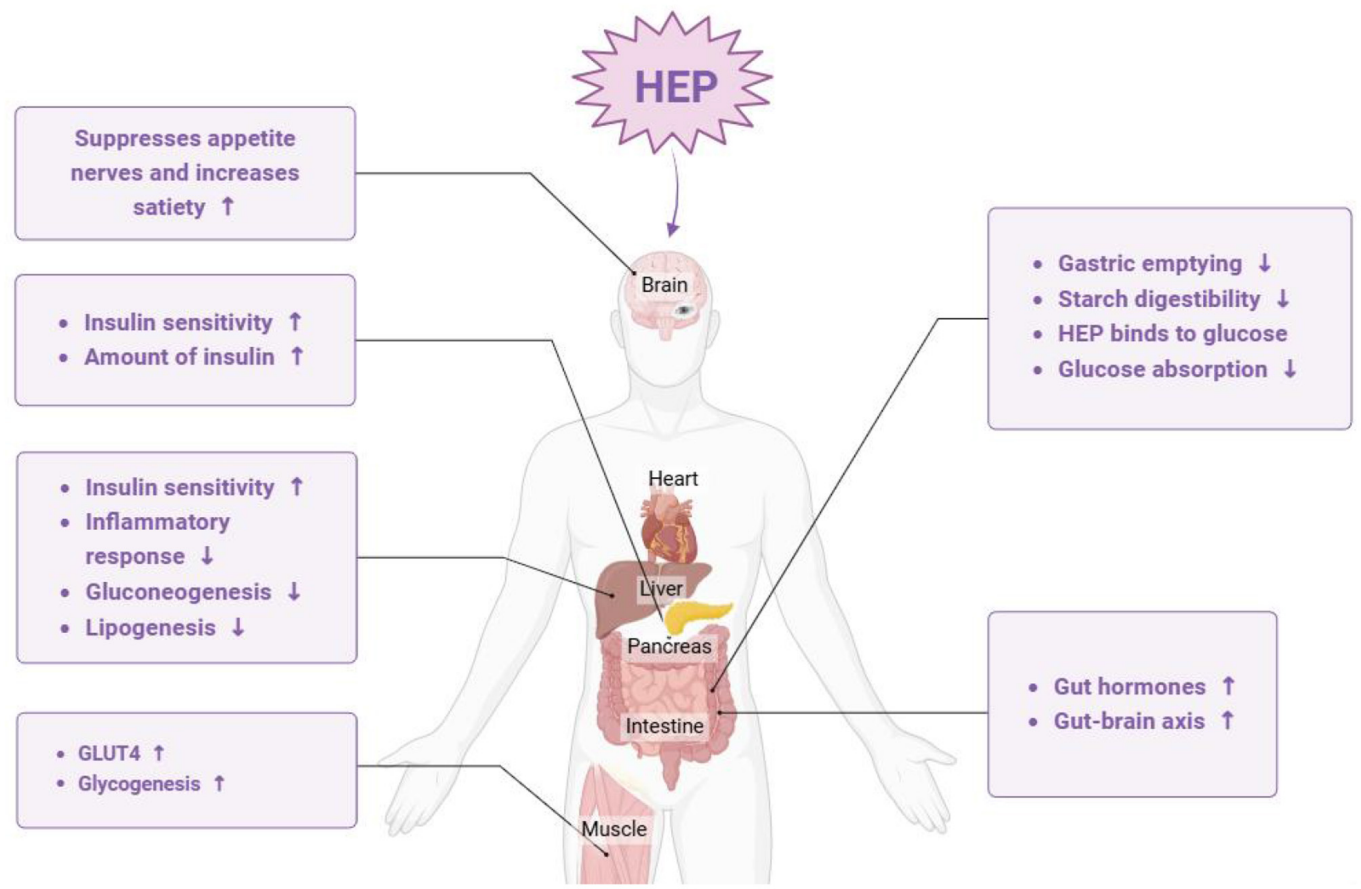
The main pathway by which *Hericium erinaceus* polysaccharide alleviates hypoglycemia (HEP: *H. erinaceus* polysaccharide).

Hyperglycemia is commonly recognized as a manifestation of diabetes, particularly type 2 diabetes, where blood glucose levels cannot be effectively regulated owing to insufficient insulin secretion or impaired cellular responses to insulin. Prolonged hyperglycemia not only contributes to the symptoms associated with diabetes but also leads to a range of serious complications. Consequently, the early detection and management of blood glucose levels are crucial for preventing the severe repercussions associated with elevated blood sugar (56, 57). Notably, the hypoglycemic potential of *H. erinaceus* polysaccharides has been investigated. These polysaccharides bind to specific receptors on cell membranes and convey information to mitochondria through cyclic adenosine monophosphate (cAMP). This mechanism enhances the activity of enzymatic systems involved in sugar metabolism, resulting in the accelerated oxidative breakdown of glucose and a subsequent reduction in blood sugar levels. Furthermore, *H. erinaceus* polysaccharides can improve both the morphology and functionality of insulin-producing cells while promoting insulin secretion. *H. erinaceus* polysaccharides have been shown to increase the utilization of glucose by peripheral tissues and target organs, including the liver and muscle (58). Together, these mechanisms enable *H. erinaceus* to significantly reduce blood glucose levels, as depicted in Figure 8. Cai et al. (59) isolated polysaccharides from the *H. erinaceus* subentity and evaluated their hypoglycemic effects, revealing that these polysaccharides effectively reduced body weight and fasting blood glucose levels, improved glucose tolerance, mitigated hepatic lesions in streptozotocin-induced diabetic rats, and positively influenced glycogen synthesis via activation of the phosphatidylinositol 3-kinase/protein kinase B signaling pathway. Furthermore, Cui et al. (60) explored the hypoglycemic mechanism of *H. erinaceus* polysaccharide. They found that *H. erinaceus* polysaccharide inhibited the progression of type 2 diabetes by inducing an imbalance in glucose metabolism, activating the adenosine monophosphate (AMP)-activated protein kinase (AMPK)/SREBP-1c signaling pathway, and increasing beneficial metabolites in the liver through the intestinal–hepatic axis. As novel dietary functional foods or therapeutic agents, *H. erinaceus* polysaccharides have considerable potential for preventing and treating diabetes as well as its complications.

### 4.7 Intestinal regulation

*H. erinaceus* polysaccharides exhibit a range of health-promoting effects by modulating the host intestinal flora composition and metabolism. The benefits of probiotics are associated with various functions, including immune regulation, anti-inflammatory bowel disease management, nervous system effects, antiobesity properties, cholesterol reduction, and inhibition of *Helicobacter pylori*. The specific effects and mechanisms underlying these activities are detailed in Table 2. Polysaccharides derived from *H. erinaceus* function by promoting the proliferation of intestinal probiotics, suppressing the growth of pathogenic bacteria, increasing short-chain fatty acid (SCFA) production, boosting intestinal immunity, and activating specific signaling pathways (61).

**Table 2:** Efficacy and potential mechanisms of *Hericium erinaceus* polysaccharides in regulating the intestinal flora.

**Effect(s)**	**Subject**	**Changes in intestinal flora**	**Change(s) in the microbial metabolites**	**Mechanisms**
Alleviates obesity and lowers cholesterol	Broilers	*Bacillus licheniformis* ↑, *Bacillus subtilis* ↑, *Lactobacillus plantarum* ↑	–	Increased the antioxidant capacity, serum total antioxidant capacity, superoxide dismutase and glutathione peroxidase levels, average daily weight gain/average daily intake, anti-inflammatory capacity, serum IL-1 receptor antagonist (IL-1ra) and IL-10 levels, and probiotic abundance; decreased malondialdehyde levels; reduced pathogenic infection (87)
	Broilers	*Lactobacilli* ↑, *Bifidobacteria* ↑, *Escherichia coli* ↓	Significantly increased the levels of SCFAs (propionic and butyric acid) in the cecum	Significantly increased mean daily weight gain; lowered serum cholesterol, triglyceride and LDL cholesterol levels; increased HDL cholesterol levels; reduced the abdominal fat percentage and liver fat content (88)
	Broilers	–	Increased the levels of SCFAs in the cecum and the levels of bile acids in feces	Reduced serum total cholesterol, triglycerides, LDL cholesterol levels; increased HDL cholesterol levels; lowered total cholesterol in the liver, leg and pectoral muscle tissues; decreased the expression of hepatic 3-hydroxy-3-methylglutaryl coenzyme A reductase (89)
Anti-inflammatory effects in bowel disease	Rats with inflammatory bowel disease	*Bacteroides* ↑, *Bifidobacterium* ↑, *Prevotella* ↑, *Parabacteroides* ↑, *Coprococcus* ↑, *Desulfovibrio* ↑, *Lactobacillus* ↑, *Corynebacterium* ↓, *Staphylococcus* ↓, *Ruminococcus* ↓, *Roseburia* ↓, *Dorea* ↓, *Sutterella* ↓	–	Significantly improved the serum levels of proinflammatory cytokines [IL-1α, IL-2, IL-8, IL-10, IL-11, IL-12, TNF-γ, TNF-α, VGEF, macrophage inflammatory protein-1α (MIP-α), and macrophage colony-stimulating factor (M-CSF)]; decreased colonic myeloperoxidase levels; improved colonic Foxp3, NF-κB p65, TNF-α, and IL-10 protein expression; reduced the abundance of proinflammatory microorganisms; increased the abundance of anti-inflammatory microorganisms; promoted the growth of *bifidobacteria* and other beneficial bacteria (90)
	Crab-eating monkeys with spontaneous ulcerative colitis	*Euryarchaeota* ↑, *Methanobrevibacter* ↑, *Candidatus_Soleaferrea* ↑, *Methanobrevibacter smithii* ↑	–	Increased body weight; alleviated diarrhea; reduced crypt abscesses and plasma cell infiltration in colonic tissues; improved intestinal inflammation and nutritional status (reduced fecal occult blood and serum C-reactive protein levels, increased serum albumin levels); remodeled the intestinal flora (91)
	Mic e with ulcerative colitis	*Proteobacteria* ↓, *Akkermansia muciniphila* ↑	Affected amino acid and glucose metabolism, and increased the content of SCFAs	Reduced the cellular and mouse colonic levels of TNF-α, IL-2β, IL-264, inducible nitric oxide synthase (iNOS), and COX-7; inhibited NLRP3 inflammatory vesicles and activation of the NF-κB, AKT and MAPK pathways; regulated the composition and metabolism of the intestinal flora (92)
Immunomodulation	Immunosuppressed mice	*Alistipes* ↑, *Muribaculaceae* ↑, *Lachnospiraceae* ↑, *Lachnospiraceae_NK4A136 _group* ↑, *Ruminococcaceae* ↑, *Ruminococcaceae*_UCG-014 ↑, *Lactobacillus* ↓, *Bacteroides* ↓, *Alloprevotella* ↓	Improved SCFAs	Significantly increased the body weight and immune organ index; modulated the intestinal flora composition; increased the abundance of SCFA-producing bacteria and the levels of SCFAs, serum immune cytokines, and key proteins in the TLR4/NF-κB pathway (TLR4 and NF-κB p65) (93)
	Normal mice	*Lachnospiraceae* ↑, *Akkermansiaceae* ↑, *Rikenellaceae* ↓, *Bacteroidaceae* ↓	–	Altered the diversity and abundance of the gut microflora; modulated immune responses through the NF-κB, MAPK, and PI3K/Akt signaling pathways (94)
	Senior dogs	*Bacteroidetes* ↑, *Firmicutes* ↓, F/B ratio ↓, *Campylobacteraceae* ↓, *Streptococcus* ↓, *Tyzzerella* ↓, *Campylobacter* ↓	–	Regulated the intestinal microecology; enhanced immunity (95)
	Healthy adults	SCFA-producing bacteria (*Kineothrix alysoides, Gemmiger formicilis, Fusicatenibacter saccharivorans, Eubacterium rectale, Faecalibacterium prausnitzii*) ↑, *Streptococcus thermophilus* ↓, *Bacteroides caccae* ↓, *Romboutsia timonensis* ↓	–	Increased intestinal flora diversity; significantly reduced serum alkaline phosphatase, LDL, uric acid, and creatinine levels (96)

VGEF, vascular endothelial growth factor; ↑, increase; ↓, decrease.

SCFAs are the end products of dietary fiber fermentation by gut microorganisms and serve as crucial links between the gut flora and the host. SCFAs can enhance the intestinal mucosal barrier, stimulate the production of immunosuppressive cytokines, and function as signaling molecules that regulate and sustain the host immune system (61, 62). Investigations into the intestinal microbiota of mice, dogs, and humans have demonstrated that *H. erinaceus* polysaccharides significantly influence microbial diversity and abundance in the intestines while increasing the prevalence of SCFA-producing bacteria. Zhuang et al. (63) reported that both the water-soluble polysaccharide (HEP-W) and alkali-soluble polysaccharide (HEP-A) from *H. erinaceus* elevated the relative abundance of key butyric acid-producing bacterial genera during fermentation. Furthermore, these polysaccharides exhibited substantial regulation of the microbial community structure while markedly increasing gas production and SCFA yields in fermentation broth. These findings suggest that *Hericium erinaceus* polysaccharide W (HEP-W) and HEP-A hold promise as potential modulators of the intestinal microbiota with higher benefits for promoting intestinal health.

Disturbances in the intestinal flora can lead to disruptions of the host's intestinal mucosal barrier and an increase in the secretion of LPS. LPS enters the circulatory system through the mesenteric vein and acts upon its target organs and tissues, resulting in intestinal inflammation and potentially inducing cancer (64). Research using experimental animal models of intestinal inflammation and other diseases has demonstrated that polysaccharides from *H. erinaceus* can ameliorate disorders of the intestinal flora. These compounds significantly increase the abundance of anti-inflammatory bacteria within the intestine, such as *Lactobacillus, Anaplasma, Bifidobacterium*, and *Prevotella*, while simultaneously decreasing the levels of proinflammatory bacteria and pathogens, including *Corynebacterium, Ruminal Streptococcus, Staphylococcus, Enterobacteriaceae, Campylobacter*, and *Shigella*, among others. Furthermore, these polysaccharides help maintain the integrity of the intestinal barrier (65). *Lactobacillus* spp. alleviate colitis induced by dextran sodium sulfate by modulating the intestinal flora and stimulating NK cells, macrophages, and T lymphocytes (66). Additionally, *Bifidobacterium shortum* has been shown to improve symptoms of colitis by reducing the levels of TNF-α, IL-1β, and IL-6 while restoring balance within the intestinal flora (67).

The gut microbiota may serve as a potential target against *H. pylori* infection (68). *H. pylori* infection is known to alter colonic pH, leading to a decrease in the abundance of the phylum Anaplasma and an increase in the phyla of thick-walled bacteria and Aspergillus. These alterations induce changes in populations such as *Vibrio desulfuricans*, Prevotella, Haemophilus, Anaplasma, *Serratia parapsilosis*, and *Enterobacteriaceae pubescens* (69). Furthermore, *H. pylori* infection can modify gastrointestinal metabolism by influencing hormone secretion, thereby affecting the composition of the gut microbiome (70). Specifically, an increase in *Lactobacillus salivarius* coupled with a decrease in *Lactobacillus gelatinosus* within the infected gut correlates with reduced gastric acid secretion (71). Additionally, polysaccharides derived from *H. erinaceus* have been shown to strongly inhibit *H. pylori* (72). Zhu et al. (73) demonstrated that Bi^3+^ combined with *H. erinaceus* polysaccharides has anti-*H. pylori* activity comparable to that of bismuth potassium citrate and also effectively reduces the risks and adverse reactions, such as epilepsy and myoclonus, associated with bismuth toxicity. Currently, reports regarding the role of *H. erinaceus* polysaccharides in combating *H. pylori* infection through regulation of the intestinal flora composition and metabolism are limited; therefore, further studies are warranted.

Due to their complex structures, polysaccharides derived from *H. erinaceus* show significant variability in their regulatory effects on the intestinal flora. Research on how *H. erinaceus* polysaccharides affect the composition of the intestinal microbiota remains in its early stages, and a definitive understanding of the relationship between the structural characteristics of these polysaccharides and the resulting changes in the gut microbiota has yet to be established. Table 3 summarizes the reported structures of *H. erinaceus* polysaccharides and their corresponding effects on the intestinal flora. The ability of these polysaccharides to modulate the intestinal flora is closely associated with their molecular weight and monosaccharide composition; furthermore, this effect is also influenced to some extent by the degree of branching within the polysaccharide chain and the various functional groups attached to them. Notably, high molecular weight polysaccharides display weaker activity than their low molecular weight counterparts do, primarily because of their limited ability to penetrate cell membranes. Chen et al. (74) isolated two distinct polysaccharides with different molecular weights from the fruiting bodies of *H. erinaceus* and investigated their gastroprotective properties in a rat model of ethanol-induced gastric ulcers. These findings indicated that the higher molecular weight β-glucan H6PC20 (2,390 kDa) exhibited superior reparative and defensive effects, whereas the lower molecular weight α-heteropolysaccharide HPB-3 (15 kDa) demonstrated stronger anti-inflammatory effects. Additionally, the content of β-(1 → 3)-glucans present within *H. erinaceus* polysaccharides may significantly influence their biological activities. The structure of *H. erinaceus* polysaccharides, characterized by a (1 → 6)-glucopyranose main chain and (1 → 3)-glucopyranose side chains, is crucial for their antitumor and immunostimulatory effects. Compared with HIPS2, which lacks these functional groups, the hypoglycemic and pancreas, liver, and kidney protective effects of HIPS1, which contains functional groups such as –NH_2_, –COOH, and S=O, were superior in streptozotocin-induced diabetic mice (75). Furthermore, the polysaccharide zinc chelate (ZnHEP), formed by polysaccharide chelation of zinc ions, demonstrated significantly higher stability and antioxidant activity than *H. erinaceus* polysaccharides. The regulatory effects of *H. erinaceus* polysaccharides on the intestinal flora are intricately linked to various factors, including their molecular mass, monosaccharide composition, type of glycosidic bonds, degree of branching, presence of functional groups, and associated complexes. However, the specific structural characteristics of polysaccharides capable of stimulating particular intestinal bacteria—and their corresponding polysaccharide profiles following hydrolysis—remain poorly understood, and this gap poses a higher level of challenges for further research in this area. Thus, it is imperative to study the conformational relationships between *H. erinaceus* polysaccharides and their regulatory effects on the intestinal flora.

**Table 3:** Sources, structures, and effects of *H. erinaceus* polysaccharides on the intestinal flora.

**Source**	**Molecular mass (Da)**	**Monosaccharide composition**	**Effects on intestinal flora**	
			**Phylum level**	**Species level**
Subentity	1.67 × 10^4^	Fuc/Man/Glc/Gal = 1.70/0.50/10.60/10.40	Firmicutes ↑, Bacteroidetes ↑, Actinobacteria ↑, Proteobacteria ↓	*Bifidobacterium* ↑, *Faecalibacterium* ↑, *Escherichia-Shigella* ↓, *Blautia* ↑, *Butyricicoccus* ↑, *Klebsiella* ↓ (97)
Subentity	4.77 × 10^3^	Fuc/Man/Glc/Gal = 1.20/1.30/23.70/0.30	Firmicutes ↑, Bacteroidetes ↑, Proteobacteria ↓	*Faecalibacterium* ↑, *Escherichia-Shigella* ↓, *Blautia* ↑, *Butyricicoccus* ↑, *Klebsiella* ↓ (97)
Subentity	8.23 × 10^5^	Fuc/Man/Glc/Gal = 0.30/1.30/9.80/0.30	Firmicutes ↑, Actinobacteria ↑, Proteobacteria ↓	*Bifidobacterium* ↑, *Faecalibacterium* ↑, *Escherichia-Shigella* ↓, *Blautia* ↑, *Butyricicoccus* ↑, *Klebsiella* ↓ (97)
Mycelium	4.60 × 10^3^	Man/Glu/Gal = 6.5/32.38/52.56	Euryarchaeota ↑	*Methanobrevibacter* ↑, *Candidatus_Soleaferrea* ↑, *Lactobacillus reuteri* ↑, *Streptococcus lutetiensis* ↓ (98)
Mycelium	8.67 × 10^4^	Glc/Gal/Ara/Xyl/Rha/Man = 76.71/14.26/4.04/2.57/1.32/1.14	Bacteroidetes ↑, Verrucomicrobia ↓, Actinobacteria ↓	*Clostridiales* ↑, *Akkermansia* ↑, *Desulfovibrio* ↑ (99)
Mycelium	9.9 × 10^3^	Fuc/Ara/Gal/Glu/Man/Xyl = 0.85/5.72/7.11/84.36/0.91/1.05	Proteobacteria ↓	*Akkermansia muciniphila* ↑ (100)

↑, increase; ↓, decrease.

### 4.8 Neurological protection

**Figure 9:**
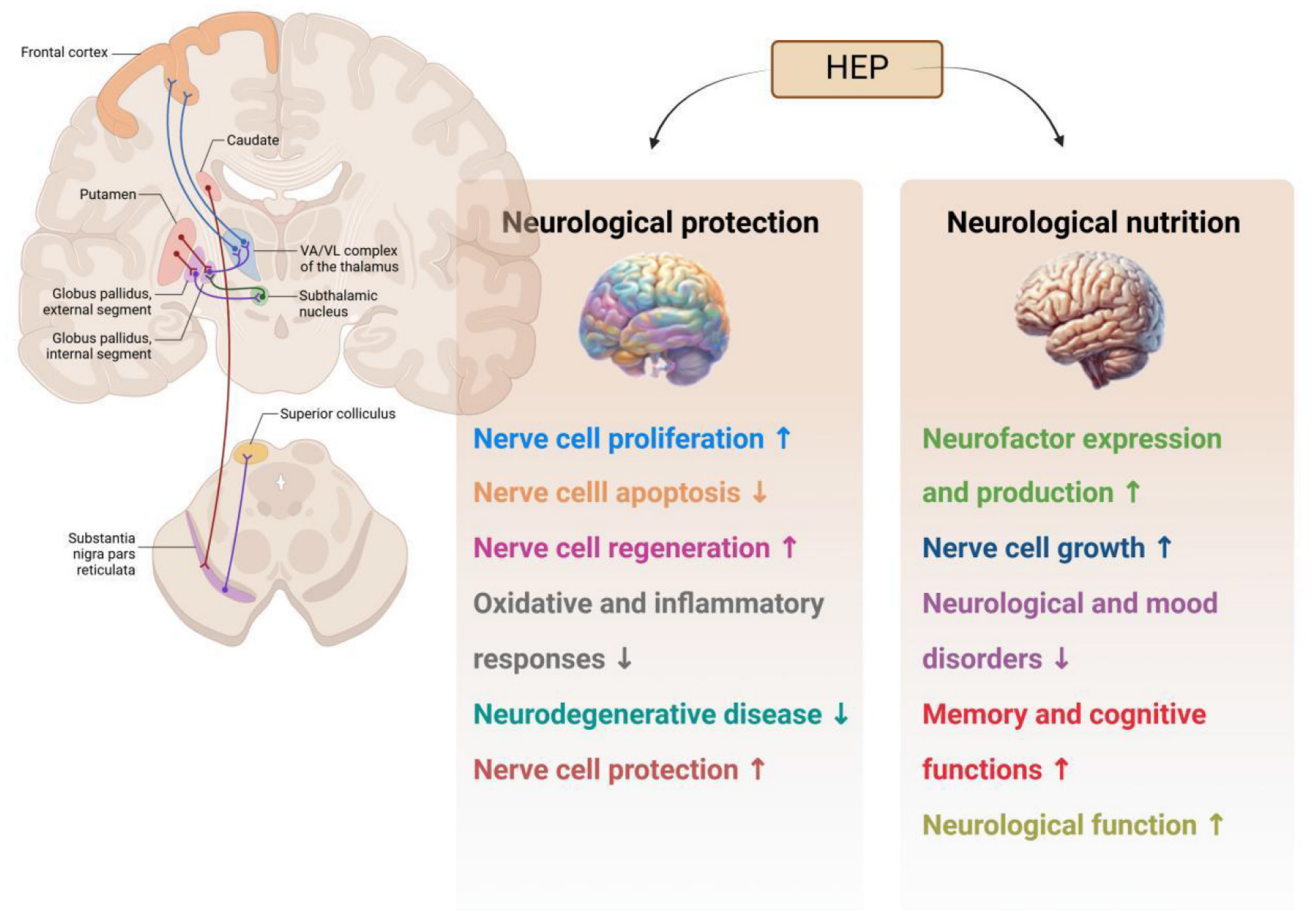
Neurological activities of polysaccharides from *Hericium erinaceus* (HEP: *H. erinaceus* polysaccharide).

Neurological protection indicates that certain substances or factors can protect neurons from injury or death. The primary objective of neuroprotection is to prevent the degeneration of nerve cells and preserve normal neurological function. In recent years, interest in the potential application of *H. erinaceus* polysaccharides for addressing various neurological and cognitive disorders has increased. Moreover, evidence suggests that these polysaccharides may exhibit neuroprotective effects, which have been attributed to their ability to support neuronal health and prevent cellular damage (76, 77). The principal mechanisms underlying the neuroprotective and trophic activities of *H. erinaceus* polysaccharides are illustrated in Figure 9. *H. erinaceus* polysaccharides promote the synthesis of nerve growth factor (NGF), inhibit β-amyloid (Aβ) cytotoxicity, and protect neuronal cells from apoptosis induced by oxidative stress or endoplasmic reticulum stress. Notable positive outcomes have been reported in the treatment of cognitive impairment, Alzheimer's disease (AD), ischemic stroke, Parkinson's disease, and age-related hearing loss (28, 78–80). Chiu et al. (81) investigated the effects of various concentrations of *H. erinaceus* polysaccharide (HEP) extracts on the toxicity to pheochromocytoma (PC12) cells induced by Aβ1–40. The accumulation of Aβ has been linked to the onset and progression of AD. Neurotoxic mechanisms associated with this condition include oxidative stress and mitochondrial dysfunction, which result in apoptosis and neuronal impairment. Researchers have reported that HEPs enhance the survival of PC12 cells under conditions of Aβ-induced toxicity. Furthermore, they noted that the ability of HEPs to scavenge free radicals and ROS increased. Consequently, HEPs protected against Aβ-induced apoptosis in PC12 cells. Hu et al. (82) demonstrated that polysaccharides isolated from the mycelium of *H. erinaceus* exhibited anti-AD activity; treatment with these polysaccharides significantly improved cognitive behaviors in AD mice while alleviating brain damage, reducing amyloid deposition, mitigating tau hyperphosphorylation, and decreasing oxidative stress within the brain. Additionally, aqueous extracts of mycelium-derived polysaccharides protected against L-glutamate-induced PC12 cell apoptosis, as did AlCl3 in D-galactose-induced mouse models of AD (83). Park et al. (84) reported that extracellular polysaccharides derived from *H. erinaceus* stimulated both the growth and axonal extension of adrenal nerve cells in a rat model. Moreover, ethanol extracts from *H. erinaceus* promoted NGF gene expression in human astrocytoma cell lines. Collectively, these neuroprotective effects suggest that polysaccharides extracted from *H. erinaceus* may represent a promising new approach for the treatment or prevention of neurodegenerative diseases (85, 86).

## 5 Conclusion and future perspectives

Polysaccharides extracted from *H. erinaceus* are bioactive macromolecules derived from the fruiting bodies, mycelia, or fermentation culture broth of this edible and medicinal fungus. These polysaccharides exhibit a variety of biological activities and have garnered increasing attention in recent years. This article summarizes the extraction and purification processes, structural properties, and biological functions of *H. erinaceus* polysaccharides and reviews advancements in research regarding their mechanisms and health-promoting applications through modulation of the intestinal microbiota and metabolism in the host. In conclusion, *H. erinaceus* polysaccharides hold high therapeutic potential and promise for future applications. However, current research faces two fundamental limitations: (1) The studies of structure-activity relationship predominantly remain at the level of apparent bioactivity, lacking molecular-level mechanistic evidence; (2) Structural modification research has focused on simple derivatives without establishing systematic function-oriented design strategies. Nevertheless, further investigations into the structures, pharmacological effects, and mechanisms underlying *H. erinaceus* polysaccharides are essential. Specifically, research efforts can be directed toward two primary aspects. First, an in-depth examination of biological mechanisms is warranted. The existing mechanism research overly relies on animal models and microbiota sequencing correlation analyses, making it difficult to distinguish between direct and indirect effects. In the future, it is necessary to consider the integrating variables of host–microbiota interactions, and combine spatial metabolomics with single-cell transcriptomics to precisely locate polysaccharides targets in the intestinal tract, and achieve cellular-resolution mechanistic insights, thus providing a scientific foundation for their application within clinical settings and drug development. Second, emphasis should be placed on both the structural modification and functional enhancement of these polysaccharides. By employing computer-aided prediction integrated with biotechnology, a targeted delivery system is developed to construct an intelligent structural and functional optimization system, thereby enhancing the bioactivity, stability, and bioavailability of polysaccharides. Ultimately, *H. erinaceus* polysaccharides modification strategies must evolve from “empirical exploration” to “precision design” to advance medical and nutraceutical applications.
